# Glial fibrillary acidic protein is elevated in preclinical AD

**DOI:** 10.1002/alz70856_105873

**Published:** 2026-01-08

**Authors:** Jane E Joseph, Eric D Hamlett, Dariusz Pytel, Steven L Carroll, Katie L Barlis, Federico Rodriguez‐Porcel, Travis E Turner, Andreana Benitez, Olga Brawman‐Mintzer, Andrew Lawson, Jens H. Jensen, Jacobo Mintzer

**Affiliations:** ^1^ Medical University of South Carolina, Charleston, SC, USA; ^2^ University of Arizona, Tucson, AZ, USA

## Abstract

**Background:**

Non‐specific Alzheimer's Disease (AD) biomarkers like glial fibrillary acidic protein (GFAP) and neurofilament light chain (Nfl) are now included in the diagnosis and staging of AD, but more work is needed to fully understand their utility in early detection of AD. The present study examined plasma GFAP, Nfl, amyloid beta (Aβ) 40, and Aβ42 in individuals with AD, mild cognitive impairment (MCI) and subjective cognitive decline (SCD) who were either amyloid positive (A+) or negative (A‐) according to florbetapir positron emission tomography scan neuroradiological read. The goal was to determine whether individuals with preclinical AD (SCD/A+) show biomarker profiles similar to those expected in AD and MCI (i.e., higher GFAP and Nfl and lower Aβ40 and Aβ42) compared to SCD individuals at lower risk (SCD/A‐).

**Method:**

Individuals with AD (24 A+, 5 A‐), MCI (21 A+, 17 A‐) and SCD (5 A+, 11 A‐), as determined by clinician referral, completed a blood draw and neuropsychological testing. Blood samples were collected, processed, and stored per previously published guidelines. Plasma samples were assayed using the Neurology 4‐Plex E+ assay on the HD‐X analyzer (Quanterix, MA). Coefficients of variation for all assays were ≤5%. Generalized linear models (GLMs) with false discovery rate correction examined the effects of diagnosis severity (AD, MCI, SCD) and amyloid status (A+, A‐) on plasma levels for each biomarker and the Aβ42/Aβ40 ratio, with covariates of age and sex. Associations between biomarkers and global cognitive functioning, as measured by the Montreal Cognitive Assessment (MoCA), were also examined.

**Result:**

The Severity x Amyloid status interaction indicated that GFAP was higher (χ^2^(2)=6.9, *p* = .032) and Aβ42/Aβ40 was lower (χ^2^(2)=13.1, *p* = .001) in AD/A+ versus AD/A‐ and in SCD/A+ versus SCD/A‐, but amyloid status did not moderate GFAP or Aβ42/Aβ40 in MCI. GLMs for Nfl, Aβ 40, and Aβ 42 did not yield significant effects. GFAP was negatively correlated with MoCA for MCI (ρ=‐.61, *p* <.001) and SCD (rρ=‐.59, *p* = .016) groups but not for AD (ρ =‐.15, *p* = .45).

**Conclusion:**

Individuals with preclinical AD (SCD/A+) showed biomarker profiles consistent with AD (higher GFAP; lower Aβ42/Aβ40) and GFAP was associated with poorer global cognition in MCI and SCD.

